# Combined endocrine approaches vs endocrine therapy alone as first line treatment in elderly patients with hormone receptor-positive, HER2 negative, advanced breast cancer: to prescribe for the patient or the physician? A meta-analysis of phase II and III randomized clinical trials

**DOI:** 10.1186/s12885-020-06933-y

**Published:** 2020-05-13

**Authors:** Claudia Omarini, Federico Piacentini, Isabella Sperduti, Monica Barbolini, Chrystel Isca, Angela Toss, Laura Cortesi, Elena Barbieri, Massimo Dominici, Luca Moscetti

**Affiliations:** 1 Division of Medical Oncology, Department of Medical and Surgical Sciences for Children & Adults, University Hospital of Modena, Via del Pozzo 71 -, 41122 Modena, Italy; 2 Department of Bio-Statistics, RCCS Regina Elena National Cancer Institute, 00144 Rome, Italy

**Keywords:** Metastatic breast cancer, Endocrine therapy, CD4/6 inhibitors, Palbociclib, Ribociclib, Abemaciclib

## Abstract

**Background:**

Elderly patients are underrepresented in clinical study where combined endocrine strategies were compared to endocrine therapy (ET) in hormone receptors positive, HER2 negative, metastatic breast cancer. The role of the new endocrine approaches in elderly women is still unclear.

**Methods:**

We performed a meta-analysis of first line phase II/III randomized trials on ET versus combined strategies considering clinical benefit and safety profile. Trials with hazard ratio (HR) for PFS in elderly patients were included.

**Results:**

Overall, the meta-analysis showed a PFS advantage for the experimental arms [HR 0.77, p 0.016] with a significant high/moderate heterogeneity [I2 65.46%, p 0.005]. For patients on CDK 4/6 inhibitors and ET, HR was 0.57 (*p* < 0.0001), with low heterogeneity [I2 0.0001%, p 0.96]. Hematological adverse events, as well as diarrhea with Abemaciclib, were significantly higher in elderly population.

**Conclusions:**

The magnitude of PFS benefit due to the combined strategies in elderly patients is similar to those reported in the overall clinical trial population. Adding CDK4/6 inhibitors to ET significantly prolongs PFS, even if toxicity profile have to be carefully considered. Future trials should be designed taking into account patients’ age, geriatric assessment and comorbidity.

## Highlights

- Due to the multi-morbidity and the drug related toxicity, combined endocrine/targeted approaches is still widely discussed in elderly patients
- The meta-analysis showed a PFS advantage for the experimental arms in patients aged ≥65 years.
- The studies with CDK4/6 inhibitors showed a significant improvement in PFS compared to endocrine therapy alone.
- The incidence of AEs in CDK4/6 inhibitors trials was significantly higher in elderly subgroup compared to younger patients.
- Treatments choose should be based on clinical benefit, toxicities profiles as well as patient preferences, needs and co-morbidities.

## Background

Advanced age is a major risk factor for breast cancer (BC), whose incidence will increase in the next years due to the increasing longevity of the population. Older women are thought to have biologically more favourable tumors, more often hormone receptor (HR) positive BC, even if is more likely to be diagnosed at an advanced stage [1]. Treatment management should not be based on age alone. Beside age, the physician decision-making process should ensure the best treatment options taking in consideration concomitant diseases, life expectancy and quality of life. Gaining evidence-based data on treatment in elderly patients without limiting their quality of life has become increasingly important. However, patients aged ≥65 years are underrepresented in most studies [2]. Establishing recommendations for management of older individuals with BC is challenging because of very limited level 1 evidence in this heterogeneous population.

In the recent years, several trials tried to associate different drugs to endocrine treatments (ET) with the aim to improve survival outcomes in metastatic HR positive, HER2 negative BC patients. Targeted and/or combined endocrine approaches such as Fulvestrant (FUL) plus aromatase inhibitors (AI), cyclin-dependent kinase (CDK) 4/6 inhibitors plus ET, bevacizumab (BEV) plus ET have been investigated as first-line treatment. In particular, multiple randomized trials showed that combining CDK4/6 inhibitors to ET, could increase the progression free survival (PFS) and overall survival (OS) in the metastatic setting, as compared to ET alone. Nevertheless, due to the multi-morbidity and the major toxicity associated with the targeted agents in elderly patients, the combination strategy is widely discussed. The present meta-analysis aimed to understand the role of the new endocrine approaches as first line treatment in the subgroup of elderly patients (aged≥65) with HR-positive metastatic BC. In particular, we focused on the studies including CDK 4/6 inhibitors, the only combination actually approved in the treatment of MBC.

## Methods

### Study objectives

We performed an analysis of the randomized phase II and III trials with the primary objective of determining the improvement in progression free survival (PFS) due to the combined endocrine strategy (such as FUL plus AI, BEV plus AI, CDK4/6 inhibitors plus ET) in comparison to standard ET alone, as first line treatment for patients aged ≥65 years with metastatic BC.

For the studies on CDK4/6 inhibitors plus ET, data on adverse events (AE) for patients aged ≥65 aged, were collected too. In particular, in each published trial, we considered the following data on AEs: grade 1–4 AEs with an incidence ≥15% and overall incidence of grade 3 and grade 4 AEs. The National Cancer Institute Common Terminology for Adverse Events (NCI CTCAE) criteria were used to grade the AEs. We evaluated the comparative risk of AEs occurrence during the combination strategy compared to ET. Moreover, the AEs accorded in the patients aged ≥65 years treated with CDK4/6 inhibitors were compared to the rate of AEs in younger ones.

### Search strategy

The systematic literature search was conducted using electronic database such as PubMed, EMBASE (from 1946), Cochrane Library (2018) and Web of Science (from 1900). We identified all the phase II and phase III randomized controlled trials, already published as full-text articles, regarding the combination strategy compared to standard ET (such as AI/TAM+/−LH-RH analogue) for the first-line treatment of patients with HR-positive HER2-negative metastatic BC. No language restriction nor restriction in terms of year of publication were applied; the final date for the database running searches was March 31st 2019. Of note, in case of multiple reports relating to the same trial, the most recently published results were selected.

The search strategy was conducted using PICO (Patient, Intervention, Comparator and Outcome) framework. The search strategy was designed by two authors (CO and LM) an approved by all the other investigators. The terms used for the research were: “breast cancer”, “endocrine therapy” and “first line therapy”. Boolean operators were used to connect specific search keywords for each database and other free text terms. Moreover, the references reported in the identified publications were checked in order to find any additional eligible trials. This meta-analysis has been conducted accordingly to the Preferred Reporting Items for Systematic Reviews and Meta-Analyses (PRISMA) and registered with the PROSPERO registration number CRD42019120215 [3]. Of note, the full protocol is freely available on the PROSPERO website.

### Article selection

Trials included in the analysis must matched the following indications:

- Phase II and phase III randomized clinical trials with published and publicly available data;
- Study population included patients with locally advanced inoperable/metastatic HR-positive HER2-negative BC;
- Trial comparing the standard first-line endocrine therapy (AI/TAM+/−LH-RH analogue) to a combined endocrine approaches;
- Studies with known information on PFS in the subgroup of patients aged ≥65 years;
- Studies with available hazard ratio (HR) and 95% confidence intervals (CI) for disease progression.

We excluded the studies with the following characteristics:

- Studies on the efficacy of new endocrine strategies but with no ET control arm;
- Nonrandomized trials;
- Phase I clinical trials;
- Studies without known data on the subgroup of patients aged≥65 years;
- Studies currently ongoing at the time of the literature search.

Data research was conducted by CO and MB. Data collected included: first author, trial’s name, year of publication, overall sample size, information on standard and experimental treatment arm, number of patients aged ≥65 years and frequency of AEs and number of patients who developed AE.

### Statistical analysis

We compared treatments using Hazard Ratio and 95% confidence intervals. Heterogeneity was evaluated by χ2 Q test and I2 statistic [4]. For the Q test, *p* < 0.05 indicated significant heterogeneity; for the I2 statistics, an I2 value > 50% was considered significant. The pooled Hazard Ratio (HR) estimate was calculated using a random-effect model [5]. Results are graphically displayed as forest plots, with HR < 1.0 indicating better outcome in the experimental arm. Publication bias was evaluated by visual inspection of funnel plots. Calculations were accomplished using the Comprehensive Meta-Analysis Software, version v. 2.0 (CMA, Biostat, Englewood, NJ, USA) [5].

## Results

**Fig. 1:**
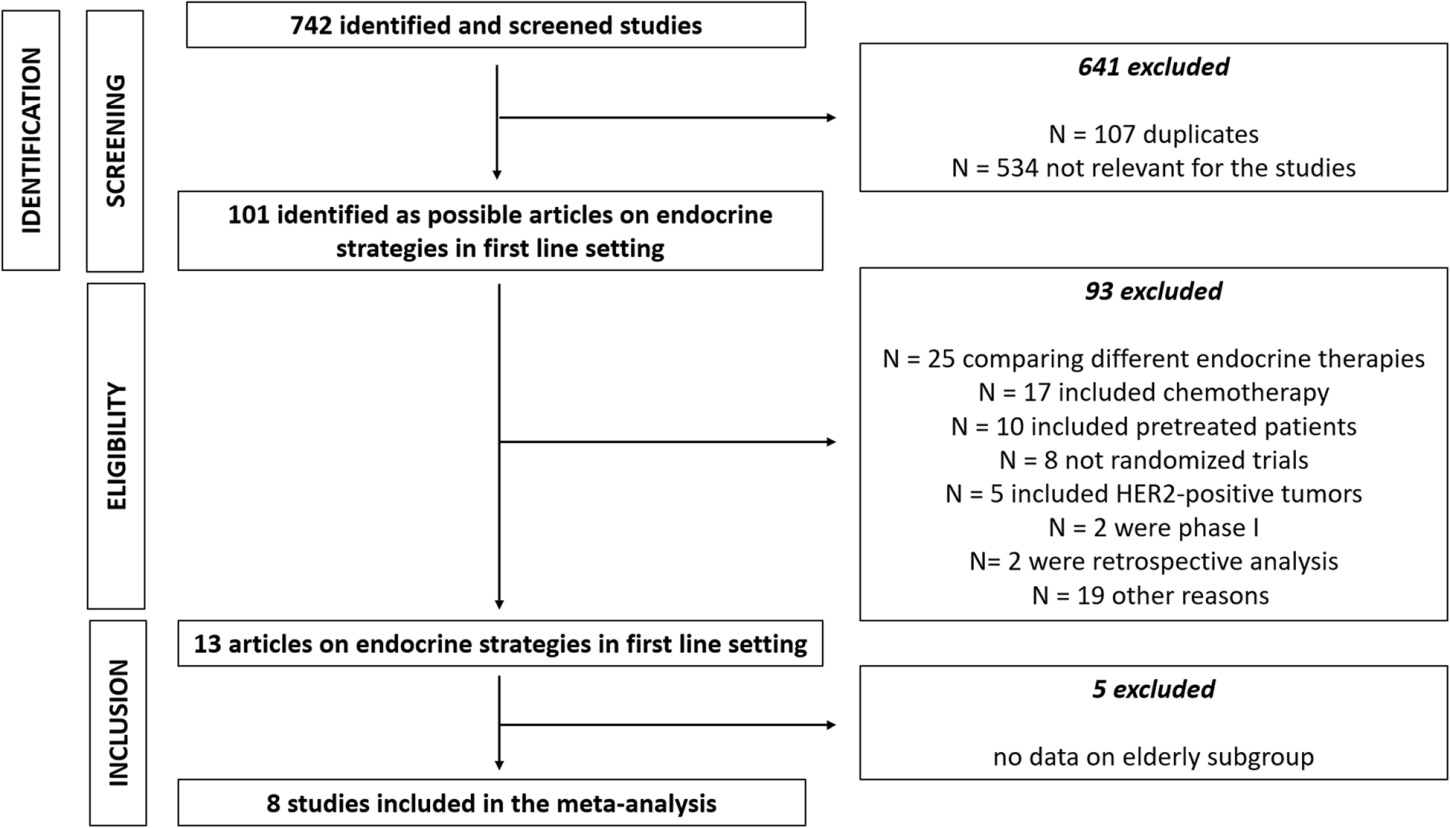
PRISMA flow chart summarizing the process to identify the eligible studies

According to our research strategy, we identified 742 publications. Based on the information found in their title and/or abstract, 101 were classified as clinical trials. Among them, 88 did not meet all the inclusion criteria (25 were clinical trials comparing different endocrine therapies, 17 studies included chemotherapy in the experimental arm, 10 trials included pre-treated patients in the study population, 8 studies were not randomized clinical trials, 5 studies included HER2-positive tumors, 2 were phase I trials, 2 were retrospective analysis and 19 articles were excluded for other reasons). Among the remaining 13 trials, 5 were excluded because data on elderly subgroup were not available (Fig. 1). Of note, SOFEA trial and ALLIANCE (CALGB 40503) trial were excluded because they considered different age-subgroup for the analysis [6, 7].

Finally, eight studies were suitable for our meta-analysis. Studies characteristics were reported in Table 1. Four trials (Paloma1/TRIO-18, Paloma2, Monaleesa2, Monarch3) investigated the role of CDK 4/6 inhibitors in association with AI [8–11], 2 trials (SWOG and FACT) analysed the combination of FUL plus AI [12, 13], while other 2 trials explored the association of ET with BEV (LEA) and Temsirolimus (HORIZON) [14, 15], respectively. In particular, the Monaleesa-2, Paloma-1/TRIO-18 and Paloma-2 trials compared, respectively, Ribociclib (Monaleesa-2) and Palbociclib (Paloma trials) in association with letrozole to letrozole alone while the Monarch-3 trial compared Abemaciclib plus a non-steroidal AI (e.g. letrozole or anastrozole) to the same AI alone [8–11]. Moreover, the LEA trial investigated the efficacy of bevacizumab added to letrozole or Fulvestrant while the HORIZON trials evaluated the combination of tensirolimus and letrozolo [14, 15]. Finally, the SWOG and the FACT trials analysed the combination of Fulvestrant plus anastrozole [12, 13].

**Table 1:** Characteristics of clinical trials included in the analysis

TRIALS	PHASE	YEAR	≥ 65 Yrs PATIENTS N.	TREATMENT ARMS	COMBINATION ARM PATIENTS N.	STANDARD ARM PATIENTS N.	PFS HR	CI
SWOG	III	2012	334	Ful + Ana vs Ana	171	173	0.79	(0.62–1.01)
LEA	III	2015	189	Bev + Let or Ful vs Let or Ful	89	100	0.82	(0.64–1.06)
FACT	III	2012	245	Ful + Ana vs Ana	134	111	1.08	(0.70–1.39)
HORIZON	III	2013	458	Tem + Let vs Let	231	227	1.21	(0.92–1.59)
MONALEESA 2	III	2018	295	Ribo + Let vs Let	150	145	0.61	(0.39–0.94)
PALOMA 2	III	2016	262	Palbo + Let vs Let	181	81	0.57	(0.39–0.84)
PALOMA 1 /TRIO-18	II	2014	76	Palbo + Let vs Let	37	39	0.50	(0.26–0.94)
MONARCH 3	III	2017	222	Abe + NSAI vs NSAI	148	74	0.57	(0.36–0.90)

*Ful* fulvestrant, *Ana* anastrozole, *Bev* bevacizumab, *Let* letrozole, *Tem* temsirolimus, *Ribo* ribociclib, *Palbo* palbociclib, *Abe* abemaciclib, *NSAI* non-steroidal aromatase inhibitor

The overall study population included 2091 patients: 1141 patients treated with the combination strategy and 950 on endocrine therapy alone. In all studies, the hazard ratio (HR) and 95% confidence intervals (CI) for disease progression was available [8–11]. Of note, AEs reported with ET +/− Palbociclib included the data of all the Palbociclib trials (Paloma1/TRIO-18, Paloma2 and Paloma3) because data on patients treated only in first line were not available [8, 9, 16].

### Comparison between experimental and control arms in patients aged ≥65 years

**Fig. 2:**
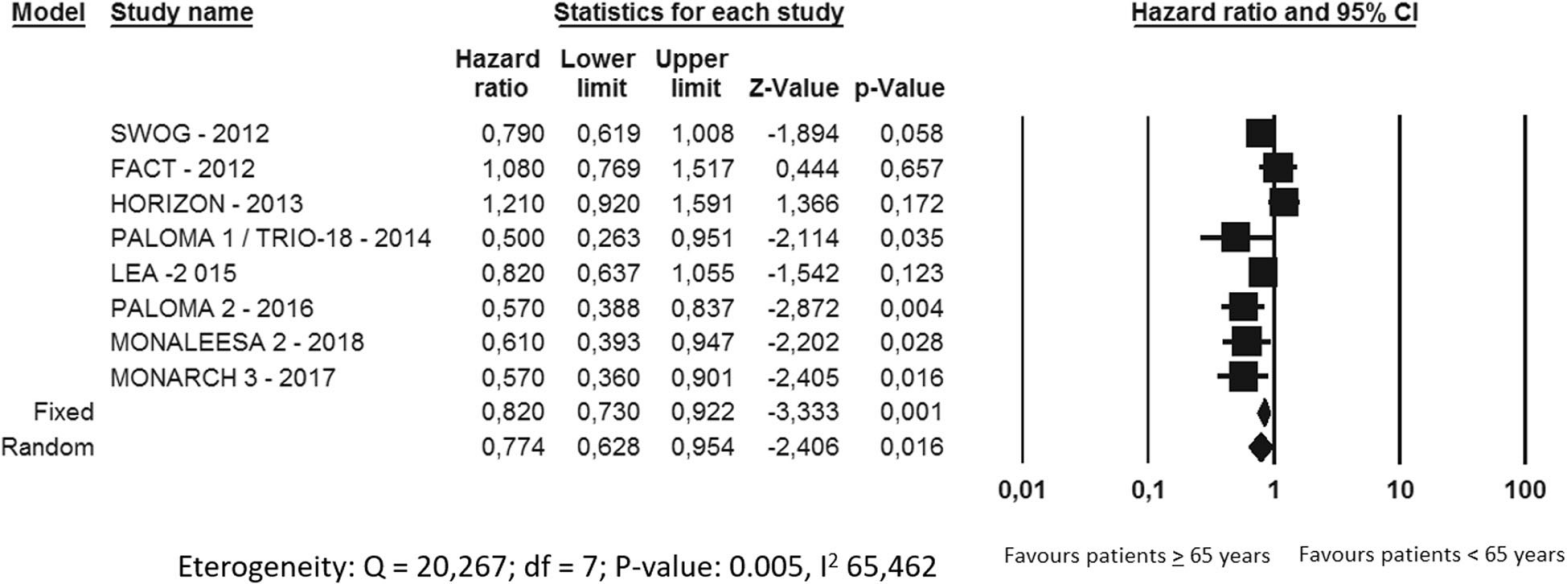
Meta-analysis of the HRs for the subgroup Elderly patients of the 8 trials included

**Fig. 3:**
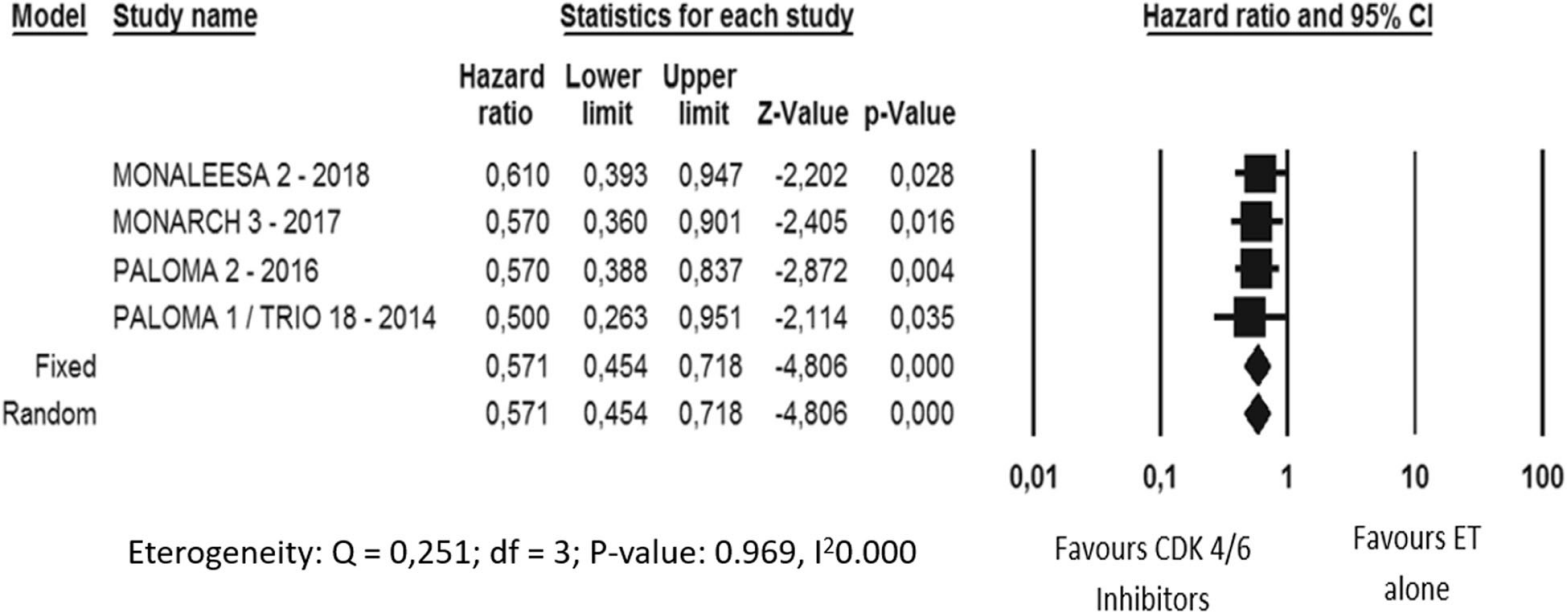
Meta-analysis of the HRs for the subgroup Elderly patients in CDK 4/6 inhibitors studies

Overall, the meta-analysis showed a PFS advantage of the experimental arms compared to the control arms (HR 0.77, 95%CI 0.62–0.95, p 0.016), with a significant high/moderate heterogeneity (I^2^ 465.46%, p 0.005) (Fig. 2). In particular, all the trials with CDK 4/6 inhibitors had a statistically significant improvement in PFS for CDK4/6 inhibitors plus ET versus ET alone (Paloma 1 /TRIO-18 HR 0.5, 95%CI 0.26–0.95 *p* = 0.035; Paloma-2 HR 0.57, 95%CI 0.38–0.83 *p* = 0.004; Monaleesa-2 HR 0.61, 95%CI 0.39–0.94 *p* = 0.028; Monarch-3 HR 0.57, 95%CI 0.36–0.90 *p* = 0.016, respectively) (Fig. 3). Regarding the other four trials (SWOG, FACT, HORIZON and LEA) no significant different in PFS between the ET and experimental strategies was found.

### *Incidence of AEs in CDK 4/6 trials* (overall incidence of grade 1–4 AEs, incidence of grade 3 and 4 AEs)

**Fig. 4:**
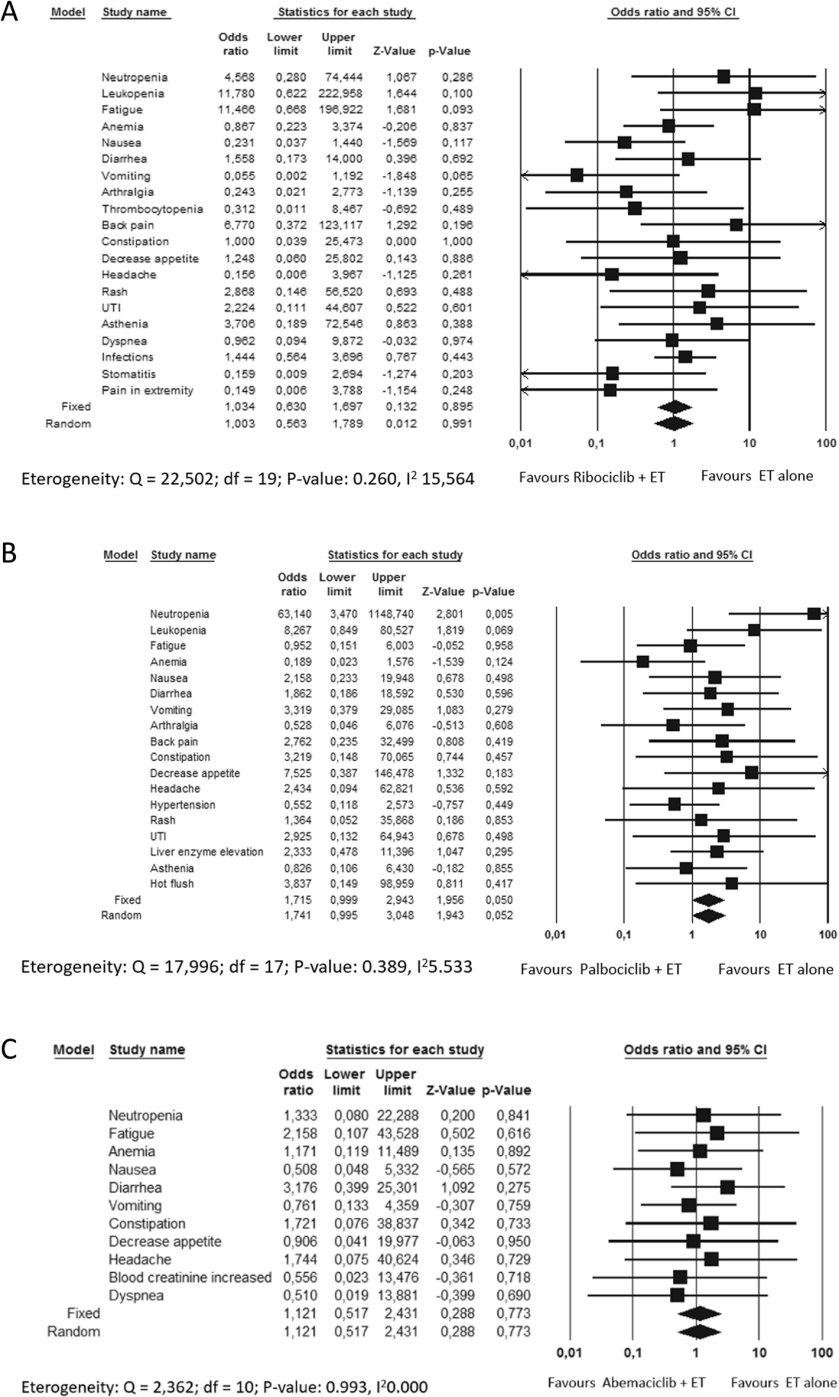
Adverse events comparative risk overview in the CDK 4/6 inhibitors trials A: Monaleesa 2 trial; B: Palama trials; C: Monarch 3 trial

In the CDK4/6 inhibitors trials the incidence of grade 1–4 AEs did not significantly differ between the experimental and the control arm (Fig. 4). Particularly, only in the Paloma trials neutropenia was the only AE significantly increased in incidence in the experimental arm.

**Fig. 5:**
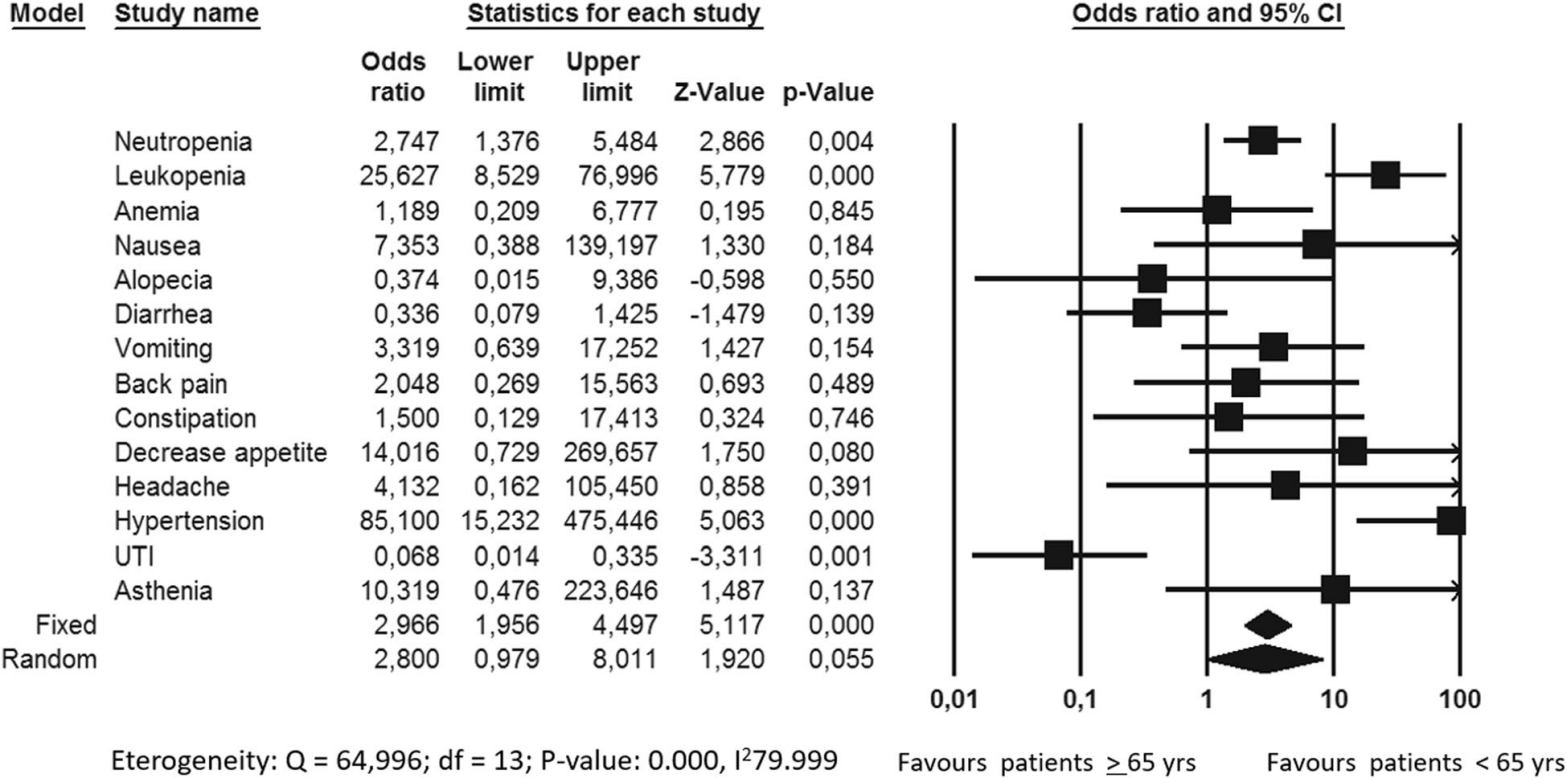
Adverse events comparative risk in patients treated with Ribociclib in Monaleesa2 trials

**Fig. 6:**
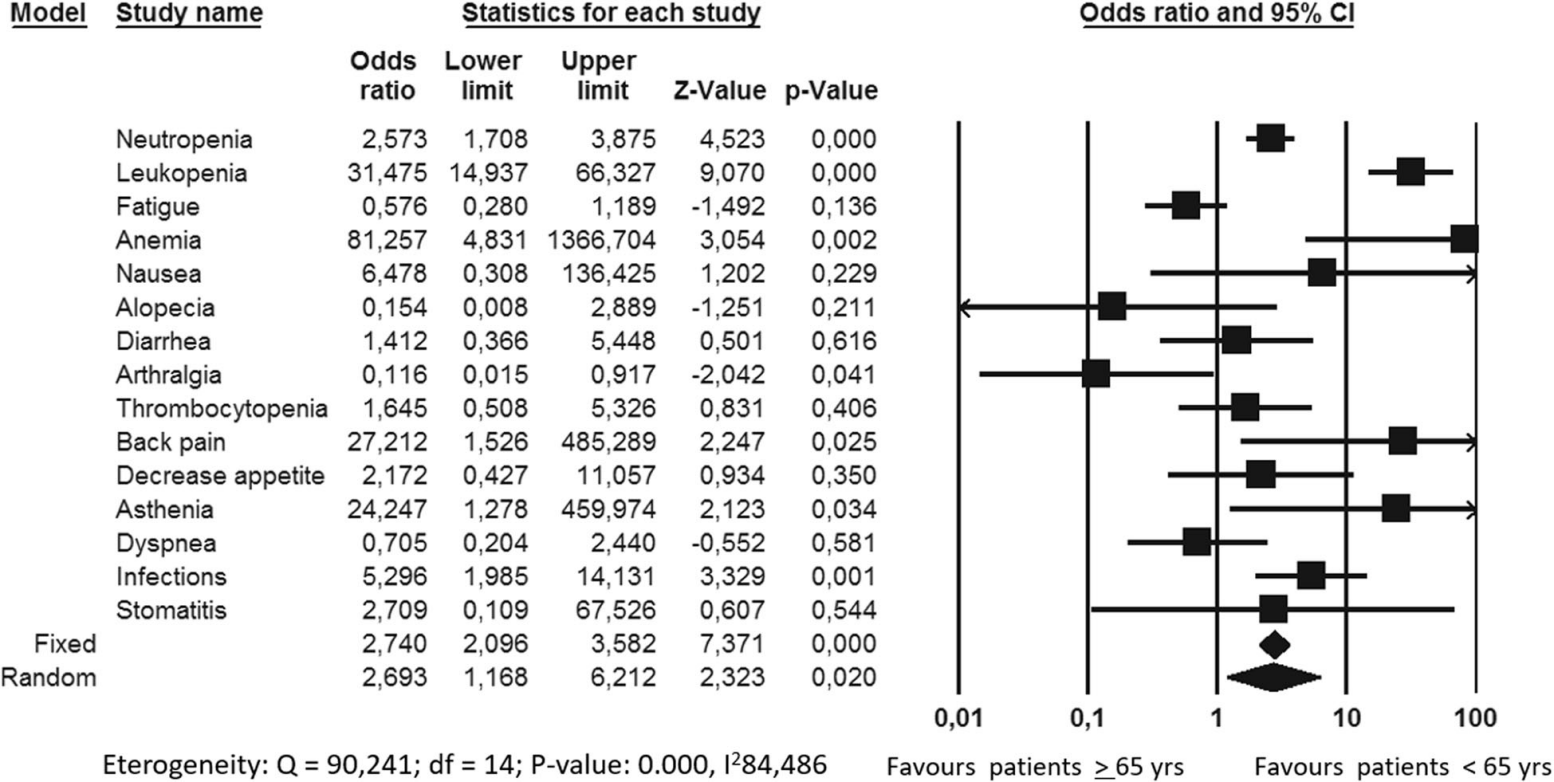
Adverse events comparative risk in patients treated with Palbociclib in all the Paloma trials (Paloma1/TRIO-18, Paloma2 and Paloma3)

**Fig. 7:**
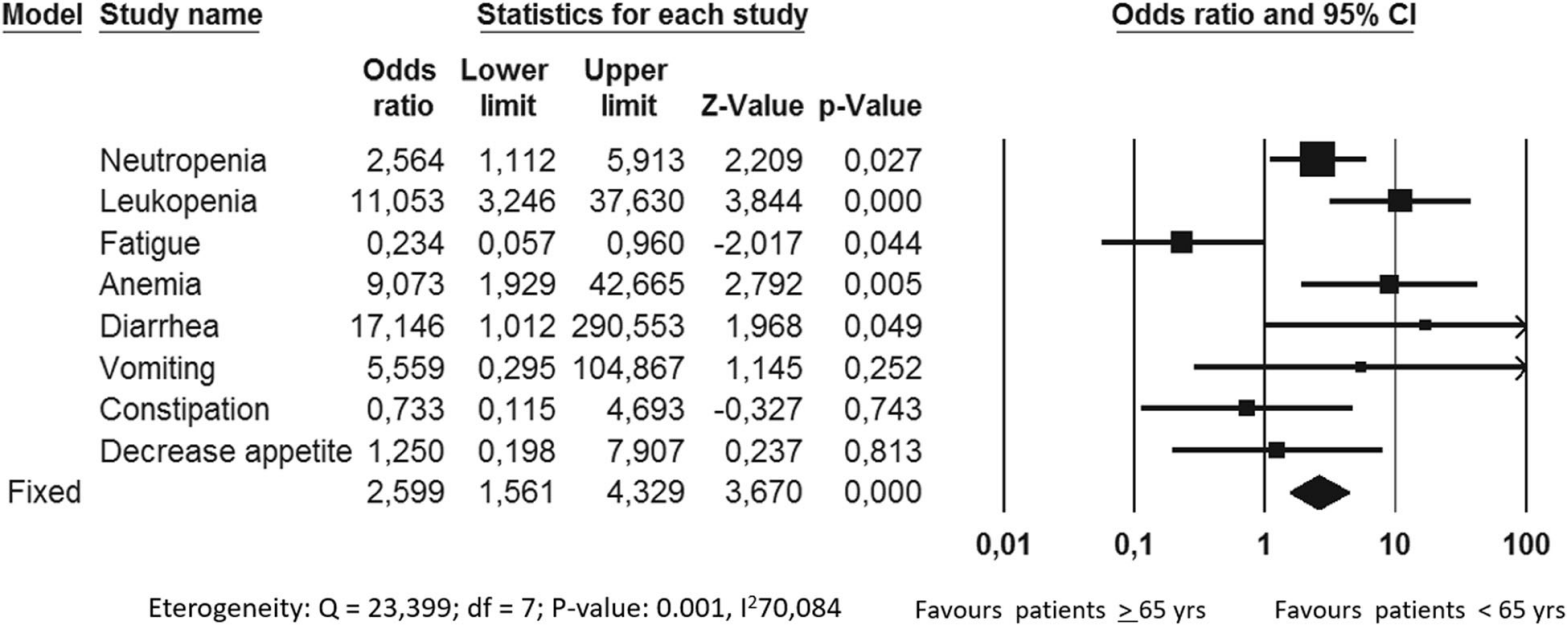
Adverse events comparative risk in patients treated with Abemaciclib in Monarch 3 trial

Overall, the incidence of AEs in Monarch3 and Paloma trials was significantly higher in elderly subgroup compared to younger patients (*p* value 0,0001 and 0,020, respectively). In particular, Abemaciclib significantly increased the incidence of neutropenia, leukopenia, anemia and diarrhea in older women while Palbociclib significantly increased the incidence of neutropenia, leukopenia, anemia, back pain, asthenia and infections in elderly subgroup (Figs. 5 and 6). Considering Ribociclib, overall there were not significant differences in AEs between the two age-based subgroups even if neutropenia, leukopenia, hypertension and urinary infections were reported significantly higher in elderly one (Fig. 7).

## Discussion

To measure the effect of a treatment in the older BC population remains an open question. Generally, the population enrolled in a clinical trial is highly selected and does not mirror the common patient of a routine outpatient setting. In the US the median age at diagnosis of BC is 62 years, 42,5% of diagnosis are made in women over 65 years; in Italy 57% of BC diagnosis are made in women over 50 years, 22% over 70 years [17]. Moreover, the life expectancy is increasing every year with 3 months, meaning an increase of 10 years in the last 40 years [18], (https://ec.europa.eu/eurostat/statistics-explained/index.php?title=Mortality_and_life_expectancy_statistics). Despite this, the accrual of older patients in clinical trials is usually difficult, up 24% of patients enrolled in metastatic protocols were aged over 65 and 13% over 70 years [19]. For that reason, the benefit of a new and expensive drugs, resulting from experimental phase III trials, are difficult to apply in the clinical daily practice.

With the aim to evaluate the impact of these new treatment strategies, we study in deep their efficacy and safety in the population over 65 years analyzing data from the phase II and III clinical trials of combined endocrine therapies compared to ET alone. Overall, in the subgroup of patients ≥65 years, combined endocrine strategies showed an improvement in PFS as first line treatment in MBC as compared to ET alone. Taken singularly, the magnitude of PFS benefit due to the addition of the CDK 4/6 inhibitors to ET was age-independent, confirming the efficacy of that new class of drugs that has changed the history of the endocrine sensitive metastatic BC [8–11]. Regarding the other four trials (SWOG, FACT, HORIZON and LEA) no significant differences in PFS between ET and experimental strategies were concluded [12–15].

Considering CDK 4/6 inhibitors, our finding are in accordance with the FDA polled analysis results where efficacy and safety of CDK4/6 inhibitors were examined in women age 75 or older [20]. The authors showed similar efficacy in older women compared with their younger counterparts, although greater serious AEs and discontinuations occurred in elderly ones [20]. Proven that the clinical benefit of CDK4/6 inhibitors is age independent, a key point prior, to initiating a treatment, is the patients’ preferences: older women may favor maintaining quality of life over prolonging survival. For that reason, AEs and patient-reported outcome (PRO) are important as well as survival benefit.

Regarding toxicity profile, we may consider that the aging process is associated with decrease in physiologic reserve of multiple systems, which may increase the risk of AEs. Moreover, possible drug interactions among concurrent medications must be take into account explicitly in older patients who are more likely to be taking concomitant drugs. In particular, co-administration of drugs that affect the function of CYP3A4 (with the consequent possible risk of reduced efficacy or increased toxicity) must be evaluate with care. In our analysis the overall incidence of grade 1–4 AEs did not significantly differed between the experimental and the control arm even if, as expected, the incidence of AE was higher in elderly population compared to younger one [8, 10, 11, 16]. Neutropenia, leukopenia and anemia were the drug related AE significantly more reported even in older population. Of note, diarrhea was higher in older patients treated with Abemaciclib compared to younger ones [11] while infections (in particular urinary infection), were significantly higher in elderly subgroup for both Ribociclib and Palbociclib [8–10, 16]. Considering PRO, the EQ-5D was the only instruments used across the PALOMA 2, MONALEESA 2 and MONARCH 3 trials. It is a preference-based measure of patient’s ability to performed daily activity. Deterioration in PRO was defined as a 1 –point drop from baseline. Data from PRO were reviewed in the FDA analysis showing that older patients, regardless the treatment arm, reported deterioration in mobility more quickly than younger ones [20].

That knowledge on clinical benefit and AEs has to lead the clinician during the treatment decision-making process in everyday clinical practice. Clinicians’ aim is to offer the best treatment option maintaining the quality of life. Physicians’ treatment choose could take into account PFS advantage, toxicity profile of each compound, preferences, needs and co-pathologies of the single patient. Evidences that come from subgroups analysis data help physician in the choice of the right treatment for the right patient. For example, in some patients with low burden of disease (as the presence of bone only metastases), older age and desire of independence, less toxic (and less expensive) drugs, such as ET, could be the right treatment option [21]. On the other hand, the clinical benefit of a new treatment could not be underestimate because of the age of the patient. Frequently, chronological age does not reflect the biological age. Elderly does not means necessarily frail; older patients must be evaluated with geriatric assessment in order to identify a better health status [10, 21]. Moreover, in order to improve the management of the new drugs some options should be evaluated. For example, dose modification or escalations should be considered. Even an unexpected toxicities, due to a mistaken dosage by the patient should be taken into account when an oral drug has going to be prescribed [10]. The multi-therapies, in presence of concomitant morbidities, represent a barrier in the choice of the new oral therapies too. Specific drug boxes in order to prevent mistaken dosage could be useful.

For that reason, in our opinion, the right way to estimate the efficacy of a treatment in the single patient has to be based on the right evidences that come from clinical trials considered representative for our target patient population. Results from clinical studies have to help clinicians to choose the most comfortable therapies for the patient and to avoid compliance problems. An increase in the effort to design clinical trials addressed to older patients with comorbidities should be taken into account. Actually, only few data are existing on frail population as well as for patients with poor performance status. Some trials are currently recruiting women over 70 years with endocrine sensitive metastatic BC to evaluate the feasibility and the role of new endocrine combined treatments [22, 23].

In spite of our data, this study presents three main limitations. Firstly, the small number of trials eligible for the analysis. Secondary, AEs reported with ET +/− Palbociclib included the data came from all the Palbociclib trials, because data on patients treated only in first line were not available. Finally, the few data on the patients reported outcomes due to lack of statistical data limited the knowledge on quality of life in elderly patients on CDK 4/6 inhibitors.

## Conclusion

In conclusion, the magnitude of PFS benefit due to the combined strategies is age-independent. In particular, the CDK4/6 endocrine combined strategies represents an active treatment option in elderly population too. Despite this, adverse events have to be carefully considered before any prescription. In the decision making process, clinician should consider and balance the risks and the benefits of the available therapies in order to offer the most effective treatment and an adequate quality of life, choosing whether to prescribe for the patient or the physician. Clinical trials addressed to older patients with comorbidities have to be encouraged.

## Data Availability

All data analyzed during this study are included in this published article.

## Abbreviations

BC: Breast cancer
ET: Endocrine therapy
HR: Hormone receptor
FUL: Fulvestrant
AI: Aromatase inhibitors
CDK: Cyclin-dependent kinase
BEV: Bevacizumab
PFS: Progression free survival
OS: Overall survival
AE: Adverse events
